# Could an artificial intelligence approach to prior authorization be more human?

**DOI:** 10.1093/jamia/ocad016

**Published:** 2023-02-21

**Authors:** Leslie A Lenert, Steven Lane, Ramsey Wehbe

**Affiliations:** Biomedical Informatics Center, Medical University of South Carolina, Charleston, South Carolina, USA; Health Gorilla, Mountain View, California, USA; Department of Cardiology, Medical University of South Carolina, Charleston, South Carolina, USA

**Keywords:** prior authorization, Clinical Quality Language, standards, artificial intelligence, appropriateness of care

## Abstract

Prior authorization (PA) may be a necessary evil within the healthcare system, contributing to physician burnout and delaying necessary care, but also allowing payers to prevent wasting resources on redundant, expensive, and/or ineffective care. PA has become an “informatics issue” with the rise of automated methods for PA review, championed in the Health Level 7 International’s (HL7’s) DaVinci Project. DaVinci proposes using rule-based methods to automate PA, a time-tested strategy with known limitations. This article proposes an alternative that may be more human-centric, using artificial intelligence (AI) methods for the computation of authorization decisions. We believe that by combining modern approaches for accessing and exchanging existing electronic health data with AI methods tailored to reflect the judgments of expert panels that include patient representatives, and refined with “few shot” learning approaches to prevent bias, we could create a just and efficient process that serves the interests of society as a whole. Efficient simulation of human appropriateness assessments from existing data using AI methods could eliminate burdens and bottlenecks while preserving PA’s benefits as a tool to limit inappropriate care.

## INTRODUCTION

Prior authorization (PA) refers to the process by which healthcare payers require providers to secure “permission” for reimbursement for a product or service prior to delivery. The PA process is thought to be essential for cost control^1^ but is perhaps the least satisfying business process for both patients and providers in our healthcare system.^2^ According to recent American Medical Association surveys, it is a major source of physician and staff burnout as well as job dissatisfaction.^3^ Patients may also be harmed by delays in access to necessary care.^4^ Experts and government agencies have called for meaningful reform.^5^ This paper reviews current informatics innovations in prior authorization and suggests an alternative approach based on the evolution of artificial intelligence (AI) technologies that, paradoxically, might also offer a more “human” approach, by allowing computer-based prior authorization to mirror assessment of the appropriateness of medical procedures performed by panels human experts.

There are several ongoing efforts to improve the prior authorization process. High-profile innovations include (1) “gold carding” providers, exempting those who have very high historical approval rates; and (2) automating the process (e-prior auth, e-PA), currently the focus of a standards based approach in Health Level 7 International’s (HL7’s) DaVinci Fast Healthcare Interoperability Resource (FHIR) Accelerator program.^6^

Among the existing options, *gold carding* is a wide-spread practice that uses a provider’s prior history of prior authorizations with a payer as the basis for a waiver of authorization requirements. This option may work best when a provider makes repeated requests to use expensive or risky therapies in the same domain, such as in cancer chemotherapy or treatment of rheumatoid arthritis or hepatitis C. One of the difficulties with the approach is the maintenance of programs in the face of evolving changes in practice. While providers can be informed of changes in approval criteria, a certain number of denials might be necessary to effect indicated changes in ordering behavior. From an informatics perspective, combining data across insurers on PA compliance using informatics standards, might be the next logical step. There would be value in creating a complete picture of a provider’s behavior toward authorization rules than a single payers view, similar to how a credit bureau manages the composite view of creditworthiness in a somewhat neutral fashion. Ethically, gold carding is challenging as receipt of gold card status may signal a transition of clinicians' judgment away from patient advocacy to compliance with payer requirements for cost-constraints. Patients have access to fewer options and those options may not be the most beneficial for them. The ethics of this approach are largely unstudied. Moreover, the patient perspective is largely excluded from the gold card approach and there are limited avenues for the system to be responsive to patients’ rights of autonomy.

The second existing practice, e-PA, focuses on developing business practices that automate the rules and reporting process of prior authorizations. Electronic communications have long been a part of the vision for prior authorizations, and current standards focus on the communication of clinical and billing information as part of the prior authorization process.^7–9^ Since the early 2000s there have been efforts to expand PA into an electronic dialogue where, in addition to transmission of billing diagnoses that might justify payment, providers could answer questions in response to a payer-specified decision logic, to automate approval. This vision has had significant technical obstacles in implementation, mostly related to payment authorization being managed by the nonclinical X12 standard. However, there has been some success with proprietary approaches and interfaces.

**Figure 1.**
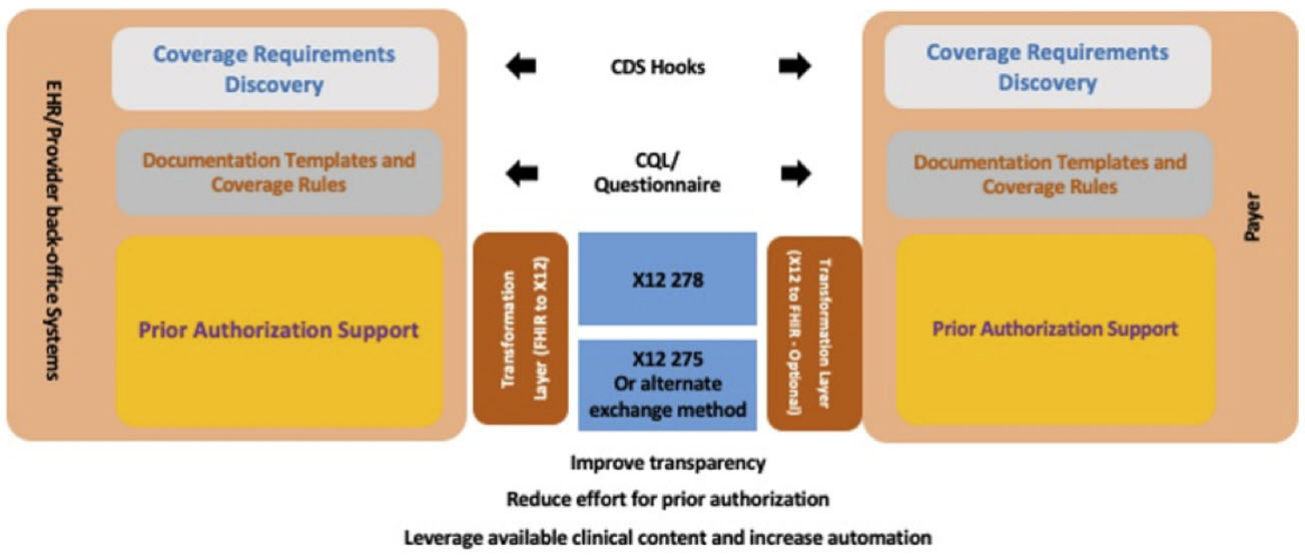
Conceptual architecture of the DaVinci Project for integration of prior authorization into electronic health records. *Source*: http://hl7.org/fhir/us/davinci-pas/STU1/index.html.

The DaVinci Project’s technical standards and implementation guides represent a substantial step forward in the effort to automate prior authorizations, leveraging both the FHIR Clinical Decision Support (CDS) Hooks and Clinical Quality Language (CQL) standards.^10^ As shown in Figure 1, reproduced from the DaVinci website, the DaVinci project recognizes two entities with differing clinical data representation languages and standards for the implementation of the exchange. Clinical providers are envisioned as using the FHIR data standards with the CDS Hooks decision support standard to evaluate cases prior to submitting requests for prior authorization. The first set of transactions trigger exchanges between the ordering provider’s electronic health record (EHR) and the payer’s system checking and validating the current coverage of an individual. The second set of transactions conveys either the rules for approval, as documented in a standardized query representation in CQL, or links the provider to a form where FHIR or other questionnaire representation standards are used to collect data. The advantage of using CQL is that the determination decision is embedded in the logic, thus making the rule transparent. However, FHIR form transmission is probably far simpler to implement and to execute, requiring only data retrieval from the EHR. The third phase of data exchange is the electronic submission of the authorization request along with all of the specified supporting clinical data. This submission, due to immense legacy investments in claims processing infrastructure and the specification of a technical standard in the 1996 Health Insurance Portability and Accountability Act (HIPAA), must be translated from an FHIR representation to the X12 278 standard or X12 275 for secure data transmission and routing to the payer for processing. There, the content may or may not be restored to the FHIR representation of data for computation for the actual authorization, as shown in Figure 1.

This process, while complex, succeeds in the replication of most of the current manual workflow for authorization. Some components are missing, such as the documentation of human review, on the payer side. There is some transparency in the approach if CQL is used for rule representation; however, this does not consistently allow an ordering provider to know the logic of the applied rule or to understand why the resultant decision is fair and appropriate. Furthermore, the solution does not provide explicit links to outside evidence sources used in the determination, nor documentation of processes supporting the rules.

## PROPOSED ALTERNATIVE

While a rule-based knowledge representation approach does reflect the current state of decision support in the EHR, it does not reflect the current state of AI tools. Rule-based methods probably are more than adequate for simple authorization decisions, such as second or third-line use of expensive pharmaceuticals, and approval of lower-cost diagnostic tests with well-structured indications. However, complex care situations with the temporality of data, evidence of success or failure of response, and positive or negative trends in clinical data items, can be difficult to represent in CQL’s rule-based format.^11^

Another challenge with the prior authorization use case is that each of the stakeholders in the decision-making process has different interests and there is no explicit way of balancing these interests one against another, except through appeal and sometimes litigation. Until processes are developed that remove inappropriate aspects of different stakeholders’ valuation of outcomes from decision-making, automation will only speed up a flawed process where the real issue is the time for human review, judgment, and conflict resolution processes.

**Figure 2.**
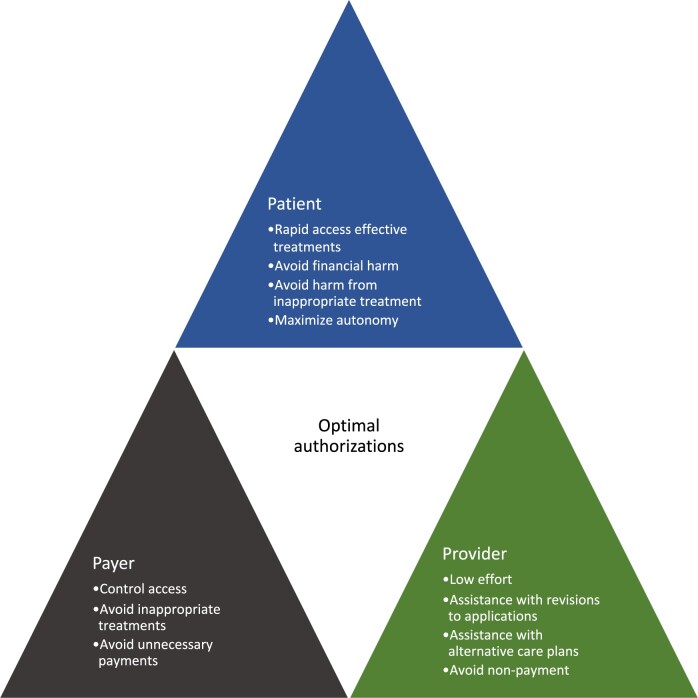
Differences in values between patients, payers, and providers in prior authorization decisions.

What would genuine “disruptive innovation” look like for PA? We believe it would be an automated approach that simulates the process of a panel of “experts,” deciding whether a test or treatment was appropriate in a specific clinical context. The panel should consider and have representatives of all three perspectives as detailed in Figure 2. Fairness and justice in these decisions should be designed and built into the framework, as a failure to do this may lead to systematic inequities, for example, based on patient demographics or other social determinants of health. Automation of expert-based PA, potentially centralized at a national level, would be a win-win-win process giving payers better information while reducing inconvenience, cost and delays for both providers and patients*.* Below, we describe such a disruptive innovation: the use of standardized data and *Human-Centric Artificial Intelligence* methods to streamline the PA process.

We propose three innovations:

1. Provider submission of an appropriately detailed and standardized set of clinical data to support ALL requests for prior authorization or authorization of direct query of the EHR for a standardized and appropriately limited set of information using FHIR data standards.
2. Use of deep learning AI methods to analyze the submitted information, along with other data available to the payer, to inform the review and approval process, with the specific goal of simulating consensus expert human judgment of the appropriateness of the product or service for the patient. AI should include explainable methodologies to ensure transparency.
3. Objective public review and certification of AI algorithms against a panel of clinical cases juried by national clinical leaders to ensure transparency and performance at the level of medical experts. The human panels used to train the algorithms should include patient representatives.

The result of our proposed innovation is that, rather than having providers submit piecemeal sets of data in response to the requirements of a rule-based system, a standardized case summary could be sent using a defined document, such as the Continuity of Care Document (CCD), as specified within the Consolidated Clinical Document Architecture (C-CDA),^12^ or a specified bundle of FHIR resources, as defined within and required by the Federally mandated FHIR^®^ standard plus the *progress note* where the treatment was ordered. While pilots could begin with the exchange of standardized data in CCD format plus the progress note, the standard e-PA data set could evolve over time to balance performance with minimum necessary principles.

Alternatively, issuing a SMART-on-FHIR security token^10^ with temporary permissions for the query of EHR-based FHIR resources, using 21st Century Cures Act EHR interface standards, could be used to authorize appropriate health records access. The clinical data, once received, would then be processed. The authorization for treatment would be determined by deep learning neural network methods, trained using consensus human judgments of actual cases, to make determinations based on analysis of the standardized clinical history of a patient. While this may sound like science fiction, the task of PA is simpler than many deep-learning neural network success stories. The resulting system would likely be more accurate and consistent than current human-mediated rule-based approaches, and better able to consider patient and case-specific factors (including data from provider notes) as well as the predicted trajectory of illness (based on comparisons to similar cases). This would also be simpler for providers, whose EHR systems would only have to send one standard set of data to any payer for prior authorization, rather than interact with or apply hundreds of rule sets based on the specific payer and the individual product or service requested.

Since 2014, deep learning (DL), a subfield of AI and machine learning based on neural networks, has achieved and even exceeded human-level performance on certain tasks in specific domains.^13^ Deep learning methodologies have since been successfully applied to several problems across medicine that were not previously feasible, particularly for the analysis of complex and unstructured data sources like imaging and free text from clinical notes. In 2017, the introduction of the transformer deep learning architectures based on the self-attention mechanism allowed for training of large language models at scale using unlabeled datasets.^14^ This was a significant leap forward in state-of-the-art deep learning-based natural language processing as it allowed for the development of large *foundational* models (eg, Bidirectional Encoder Representations from Transformers [BERT]^15^ and Generative Pretrained Transformer 3 [GPT-3]),^16^ pretrained on massive amounts of text data from the Internet. There are now several publicly available Transformer-based language models adapted to the clinical domain through further training on biomedical corpora (eg, ClinicalBERT,^17^^,^^18^ GatorTtron,^19^ Clincal-Longformer^20^). A frequently cited obstacle to creating AI systems is access to labeled data. However, these foundational models have learned robust semantic representations of text. They can be fine-tuned for specific tasks with relatively few labeled examples (even as few as 100 or less) using techniques such as transfer learning^21^ or meta-learning,^22^^,^^23^ and still achieve state-of-the-art performance.

While training foundational models is computationally demanding, adapting an existing clinical foundational model for the PA domain via fine-tuning is a relatively modest computational task depending on the training dataset size (on the order of 15–20 days using a single graphics processing unit instance [eg, NVIDIA RTX A5000 or equivalent] for a corpus the size of MIMIC-III^24^ [∼112 000 clinical notes])^25^^,^^26^. Given that routine care generates many millions of PA decisions a year, developing an initial training set and base model for PA adjudication is an achievable prospect. This base model could then be further fine-tuned with minimal computational expense (on the order of minutes to hours of computational time)^27^^,^^28^ for specific PA determinations based on smaller labeled datasets that reflect consensus judgments of expert panels.

Furthermore, it has been observed that Transformers can learn new tasks without any additional training by prompting the model with only a few examples at inference time, a process called *few-shot learning.*^29^ As the technology evolves, this capability could further increase generalizability and reduce the computational burden of adapting to particular PA use cases. Importantly, while training of such a network may be computationally intensive, applying the resultant algorithms to make predictions could happen almost instantaneously. Transformer architectures can additionally combine structured data, such as diagnosis codes or laboratory results with free text clinical notes in a *fusion* or data agnostic framework, as well as account for the sequential structure or temporality of data observations to improve predictions based on temporal trends.

**Figure 3.**
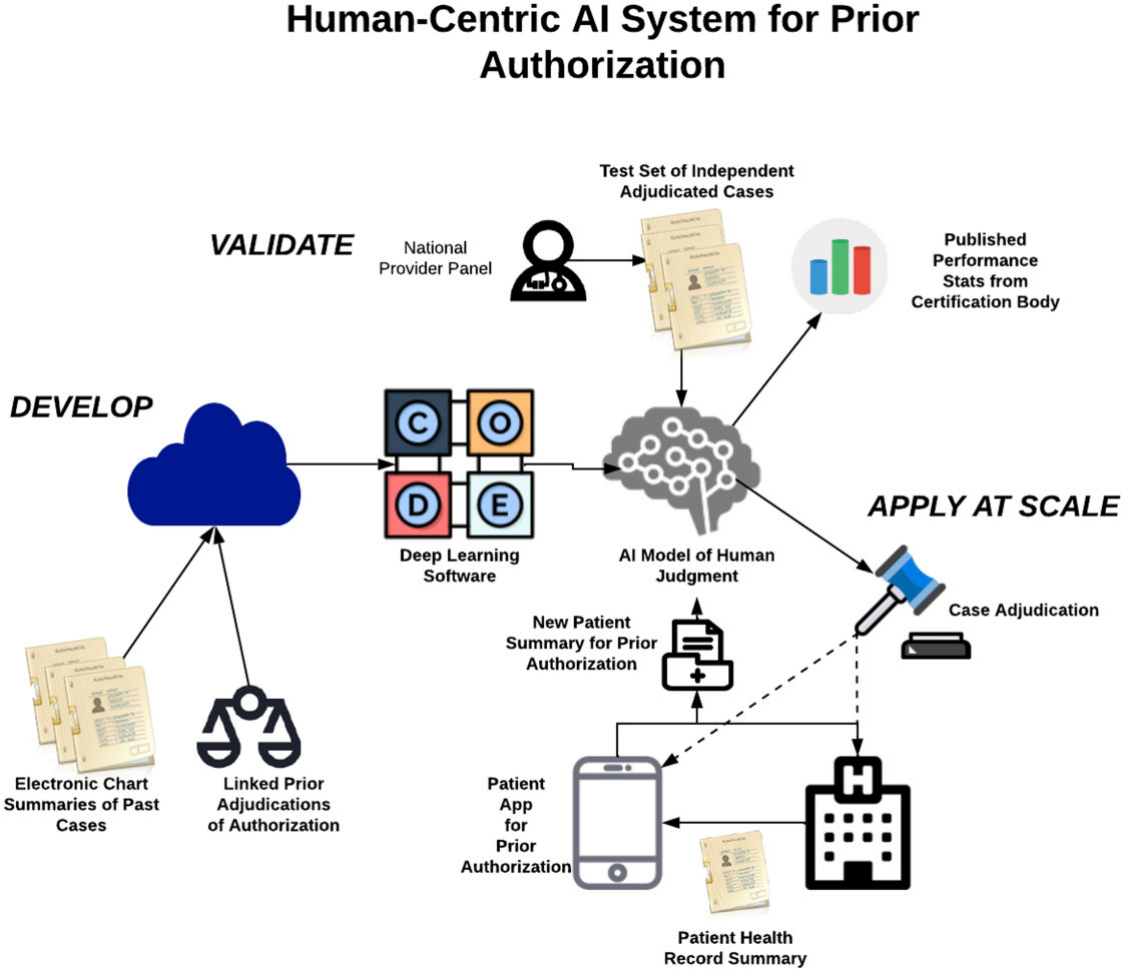
Proposal for a human-centric AI system for prior authorization. AI: artificial intelligence.

We believe this AI-driven approach could improve the PA process, by providing an initial standardized, objective review, reducing errors and improving efficiencies. When combined with a human-based oversight and appeal process, it could also be, paradoxically, more “human-centric” than the current process, which involves humans or machines constrained to following a standard set of rules until deep in the appeals process. Unlike these rules-based methods, an AI approach allows for direct analysis of more complex, unstructured data using explicit supervisory signals to replicate the refined judgment of a consensus of experts and stakeholders. Figure 3 shows a conceptual representation of our approach with human review of training cases, a certification body for AI systems, and integration of apps for patients to keep them informed and to contribute additional data as needed.

## ADDRESSING POTENTIAL BIAS IN AI MODELS

Implementation of AI approaches for PA may require a staged approach because of the potential for bias in AI models.^30^ One of the most important concerns may be a lack of representativeness of training sets.^31^ Existing foundation models could be biased by a low prevalence of minority group members or the absence of populations with a lack of access to care.^32^ Without careful development, biases in generative data sets could enshrine existing issues in resource allocation in healthcare systems.^31^^,^^33^^,^^34^ Existing foundation models should be used to demonstrate the feasibility of the approach and its potential performance. If AI-based efforts prove successful, a national effort to curate a truly representative healthcare data set may be required. In addition, the use of race, gender, age, and social determinants of health in DL algorithms for PA would need to be carefully reviewed, as inclusion can have unintended consequences.^35^ The use of human panels in developing final training data sets may reduce some types of bias. Even so, the potential for occult biases should be continually re-examined through periodic reviews of performance as part of governance.^36^

## CONCLUSIONS

Topol has argued for the opportunities and merits of combining AI with human reasoning to refocus medical practice.^37^ We propose to extend this to administrative medicine. In this case, the AI approach for more complex adjudication would simulate composite human judgment (the art of medicine as well as science). The algorithms’ performance standard would be decision-making at the level of a national expert panel, with the cases used for validation reviewed by accrediting bodies, and with patient input, helping to ensure judgments are appropriate. The algorithms also could be more just, validated to be neutral to socioeconomic status, race, gender, age, and other social factors except insofar as these factors represent risk factors for, or change the effectiveness of treatment. The authors of this proposal are not aware of any aspect of the administrative healthcare system that has been engineered using the latest technology to make that system efficient, empowering, and just. However, we believe is time that we begin to make administrative medicine more efficient and more human through the application of AI.

## Data Availability

Not applicable.
